# Insights into extrinsic foot muscle activation during a 75 min run using T2 mapping

**DOI:** 10.1038/s41598-021-86810-1

**Published:** 2021-04-01

**Authors:** Grischa Bratke, Steffen Willwacher, Florian Siedek, David Maintz, Daniela Mählich, Kilian Weiss, Tilman Hickethier, Gert-Peter Brüggemann

**Affiliations:** 1 Department of Diagnostic and Interventional Radiology, University of Cologne, Kerpenerstraße 62, 50937 Cologne, Germany; 2 Institute of Biomechanics and Orthopaedics, German Sport University Cologne, Am Sportpark Müngersdorf 6, 50933 Cologne, Germany; 3 Philips Healthcare Germany, Röntgenstraße 24, 22335 Hamburg, Germany; 4 Radiologie Heinrichsallee, Heinrichsallee 50-54, 52062 Aachen, Germany; 5 True Motion Running GmbH, Hermann-Sudermann-Str. 3, 48155 Münster, Germany

**Keywords:** Skeletal muscle, Magnetic resonance imaging, Medical research

## Abstract

The extrinsic foot muscles are essentially for controlling the movement path but our knowledge of their behavior during prolonged running is still very limited. Therefore, this study analyzed the time-course of muscle activation using T2 mapping during 75 min of running. In this prospective study, 19 recreational active runners completed 75 min of treadmill running at a constant speed. Interleaved T2 mapping sequences were acquired and segmented at timepoints 0, 2.5, 5, 10, 15, 45, and 75 min. ANOVA for repeated measurements followed by a Tukey post hoc test and Pearson correlation between running speed and initial signal increase at 2.5 min were calculated. All muscles showed a significant signal increase between baseline and 2.5 min (e.g. medial gastrocnemius: + 15.48%; *p* < 0.01). This was followed by a plateau phase till 15 min for all but the extensor digitorum longus muscle and a significant decrease at 45 or 75 min for all muscles (all *p* < 0.05). Correlation between running speed and signal increase was negative for all muscles and significant for both gastrocnemii (e.g. medial: r =  − 0.57, *p* = 0.0104) and soleus (r =  − 0.47, *p* = 0.0412). The decrease of relaxation times times in the later running phases was less pronounced for faster runners (≥ 10 km/h). T2 relaxation times do not only decrease after cessation of exercise but already during prolonged running. The lesser initial increase and later decrease in faster runners may indicate training induced changes.

## Introduction

Millions of recreational runners aim for longer distances such as marathons by pushing their limits with a relatively high risk for injuries. Van Middelkoop et al. reported that 28% of runners suffer an injury in the month before or during a marathon^1^ with most running associated injuries located in the knee or lower leg with 41.7% and 27.9%, respectively^2^. The main reasons for injuries are training errors and overuse (about 72%)^3^ followed by extrinsic and intrinsic risk factors such as previous injuries, body size, anatomical alignment, and level of competition^4^.

Running is a complex interaction of activation and relaxation of the involved muscles, starting with muscle-tuning even before heel strike^5^. The central nervous system gains input over the ground reaction forces and tunes the muscles accordingly to a predefined and cost-effective personal preferred motion path^6^. The extrinsic foot muscles (gastrocnemius medialis, gastrocnemius lateralis, soleus, tibialis anterior, tibialis posterior) play an essential role in controlling the movement path as they act as foot invertors and prevent pronation of the foot during running. Electromyography is a widely used technique for the detection of muscle activation. However, surface EMG is only suitable for assessing large muscle groups without good spatial resolution, and even the invasive method of intramuscular EMG is still affected by "cross-talk" between closely adjacent muscles^7^. Furthermore, both methods are highly dependent on the placement of the electrodes^8^. Another non-invasive way of muscle analysis is by means of ultrasound. The focus here is on morphological changes, which, however, can only be determined focally in the context of a defined movement for individual muscles or as long-term changes^9–11^.

In 1988, Fleckenstein et al.^12^ were the first to show a correlation between activation patterns of skeletal muscles before and after exercise with an increase of the T2 signal using magnetic resonance imaging (MRI). Using MRI allows near real-time, non-invasive observation of muscle activation with excellent spatial resolution. Although the exact physiological mechanism remains unclear it is believed that most of the effect is due to intracellular events with an increased microcirculation in the active muscle, accumulation of osmolytes (phosphate, lactate, and sodium) and intracellular acidification resulting in increased intramuscular water content and volume of the muscle^13–15^. Multiple studies were able to detect muscle activation in lower extremities using T2 mapping scans after exercise, resulting in increased relaxation times^13,16–18^ and showing good correlation between muscle activation and exercise intensity^19–21^ as well as glucose uptake^22^. Jenner et al. reported relaxation times increasing to a maximum after only a few repetitions of dynamic ankle dorsiflexion exercise with a following plateau phase for the next 15 min^18^ and Fisher et al. described a return to the initial level about 20 min after cessation of the exercise^23^. While the existing literature covers direct pre- and post-exercise comparisons and the starting phase (first 15 min), we still lack information about the reaction and adoption of muscle activation to prolonged running.

Therefore, this study is the first to describe the time-course of muscle activation based on T2 mapping during a prolonged run of 75 min to test for the hypothesis of a consistent plateau phase after initial activation.

## Materials and methods

### Participants

**Figure 1:**
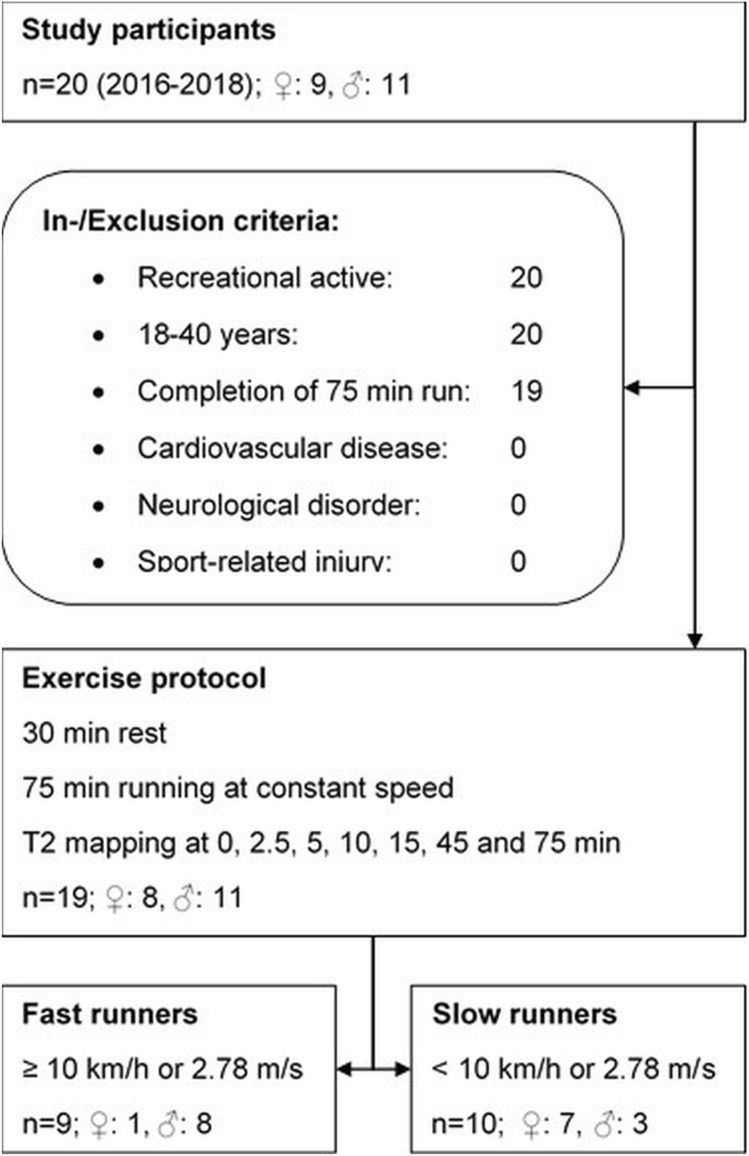
Flow diagram for study participants with exercise protocol and subgroup analysis.

This prospective study was approved by the local institutional review board of the University of Cologne and registered in the German Clinical Trials Registry (DRKS00011152) on the 02/07/2018. Informed consent was obtained from all study participants and all procedures were carried out in accordance with relevant guidelines. Participants and corresponding imaging data were acquired from December 2016 until August 2018. Participants needed to be recreationally active, between 18 and 40 years old and without any known neurological disorder, cardiovascular disease, or sport-related injuries at the time of the study (Fig. 1). Dividing the subjects in terms of running speed was based on the previously described correlation between load intensity and muscle activation^19–21^. The 10 km/h were chosen in order to obtain two groups as equal as possible.

### Exercise protocol

Each volunteer tested and subsequently chose the highest possible running speed that he or she was able to maintain for 75 min on the provided treadmill (F85, Sole Fitness, Neu-Ulm, Germany). The treadmill was positioned directly in front of the scanner room to minimize time and potential influences between running and scanning. Based on the results from Fisher et al.^23^, all volunteers completed an initial resting phase of 30 min on the moveable MR table outside the scanner room to neutralize any exercise or muscle activation before the experiment. The volunteers laid supine on the table in a relaxed position with slightly bent knees (about 20°) and were brought to the MR room to avoid any movement of the leg before starting the baseline scan. The running phases were divided into different sections with interposed MR scans to allow a high temporal resolution of muscle activity changes. The measuring timepoints were 0, 2.5, 5, 10, 15, 45 and 75 min to account for the previously described rapid increase of T2 times and the following plateau. The effective times for MRI scanning, running on the treadmill, and switching between the scanner and the treadmill were recorded using a stopwatch. All runners were asked whether they felt exhausted or could continue running with the same running speed.

### MRI examination

All scans were performed on a whole-body 3.0 T MRI system (Philips Ingenia 3.0 T, Philips Healthcare, Best, the Netherlands) equipped with a dedicated 16-channel transmit and receive knee coil. To ensure full consistency between all scans for the individual participant, a custom-made MR-compatible board fixed to the MR table was used to position the feet at the same spot after each bout. For each participant, the coil base was kept fixed to the MRI table while the right calf was positioned in the center of the coil base. The initial scan included T1-weighted sequences in coronal and axial direction for optimal anatomical planning. A Carr-Meibom-Purcell-sequence identical to the one described by Willwacher et al.^24^ and comparable to Schuermans et al.^25^ was used to obtain T2 maps after acquisition of 16 echoes ranging from 10 to 160 ms with a repetition time of TR = 2000 ms (flip angle: 90 degrees, SENSE factor: 1.7, voxel size: 1.5 × 1.5 × 5 mm). The relaxation time was calculated by the scanner based on the exponential decay of the signal intensity. Ten slices with a 15 mm gap were acquired, resulting in a FOV with a craniocaudal length of 185 mm starting from the fibula head and with a transversal dimension of 150 × 197 mm.

### Data analysis

Due to field inhomogeneities or a poor signal-to-noise ratio in some scans, the first and last two slices were excluded with six slices remaining for analysis. On each slice and for all time points the medial (MG) and lateral gastrocnemius (LG), soleus (SO), peroneus longus (PER), tibialis posterior (TP), tibialis anterior (TA) and extensor digitorum longus (EDL) were manually segmented by a radiologist specialized in musculoskeletal imaging (7 years of experience) using HOROS viewer 3.3.2 (The Horos Project, Annapolis, MD, USA). Segmentation was performed on the first echo (TE = 10 ms) due to the best delineation of anatomical structures and copied to the calculated T2 map. Comparable to previous studies, vessels and intramuscular fat were excluded^25^. The average T2 relaxation time for each muscle for a given time point was calculated as the sum of multiplying the relaxation time for each slice with the cross-sectional area (CSA) averaged to the whole measured cross-sectional area of the muscle (ACSA), comparable to previous work by Ploutz-Snyder et al.^13^: ${T2 \,relaxation \,time}_{muscle}=\sum_{slice \,1}^{slice\, 6}{relaxation\, time }_{slice \,x}\times \frac{{CSA}_{slice \,x}}{{CSA}_{all \,six\, slices}}$

This procedure ensures that the relaxation time for each slice is included according to the total percentage of muscle volume. Muscle volumes for 18 of the volunteers in this study had been reported previously by Wilwacher et al.^24^.

### Statistical analysis

Statistical analyses were performed using GraphPad Prism Version 8.4.1 (GraphPad Software Inc., San Diego, California, USA). All data are reported as the mean ± standard deviation (SD). The data was checked for normal distributaion using the Kolmogorov–Smirnov test. The continuous data of the relaxation times were tested for significant differences with a one-way analysis of variance (ANOVA) for repeated measurements followed by a post hoc analysis using the Tukey test to correct for multiple comparisons. The times for the individual running, changing, and measuring segments were tested using ANOVA. Correlations of the running speed with the increase in relaxation times between the baseline and after the initial increase at 2.5 min were assessed by calculating Pearson’s correlation coefficient. Differences between fast and slow runners were assessed with the unpaired *t* test. A *p* value of < 0.05 was considered statistically significant.

## Results

Eleven male and nine female healthy young volunteers participated in this study (Table 1). One female volunteer was unable to complete the 75 min run at the chosen speed and was excluded from the following data analysis (n = 19). The average running speed was 9.66 ± 1.40 km/h or 2.68 ± 0.40 m/s, and all runners felt exhausted, fulfilling the criteria for maximal possible running speed over the 75 min period. Normality test was passed for all timepoints with all *p* > 0.1.

**Table 1:** Demographics of study participants before and after dropout of one volunteer due to an incomplete run as well as subgroups for fast (≥ 10 km/h or 2.78 m/s) and slow (< 10 km/h) runners. The subgroups differed significantly with regards to height, sex and speed (marked with *).

	n = 20	N = 19	Fast	Slow
Age [years]	28.75 ± 3.18 (23–33)	28.68 ± 3.25 (23–33)	29.33 ± 2.49	28.10 ± 3.70
Height [cm]	176.10 ± 7.94 (162–198)	176.42 ± 8.02 (162–198)	180.56 ± 9.74	172.70 ± 2.72*
Body mass [kg]	70.20 ± 9.74 (51–95)	70.74 ± 9.70 (51–95)	73.33 ± 11.16	68.40 ± 7.43
Sex	11 male/9 female	11 male/8 female	8 male/1 female	3 male/ 7 female*
speed [km/h]	2.68 ± 0.39 (2.08–3.61)	2.68 ± 0.40 (2.08–3.61)	3.04 ± 0.25	9.66 ± 1.40*

The effective time for the individual running sessions were almost identical to the targeted times with 2:31,18 ± 0:01,58, 5:00,99 ± 0:03,02 and 30:01,97 ± 0:03,27 min for the 2.5, 5, and 30 min running segments, respectively. The running sessions were interrupted in total by 3:45,70 ± 0:46,40 min consisting of the average time within the scanner with 2:17,23 ± 0:27,54 min, 0:48,40 ± 0:15,87 min when switching from the scanner to the treadmill and 0:41,23 ± 0:07,95 min for switching from the treadmill back to the scanner. The time from leaving the treadmill until the end of the scan as a potential confounding factor for the relaxation times ranged from 2:55,55 to 3:04,23 min without significant differences between the individual running segments (all *p* > 0.05).

**Figure 2:**
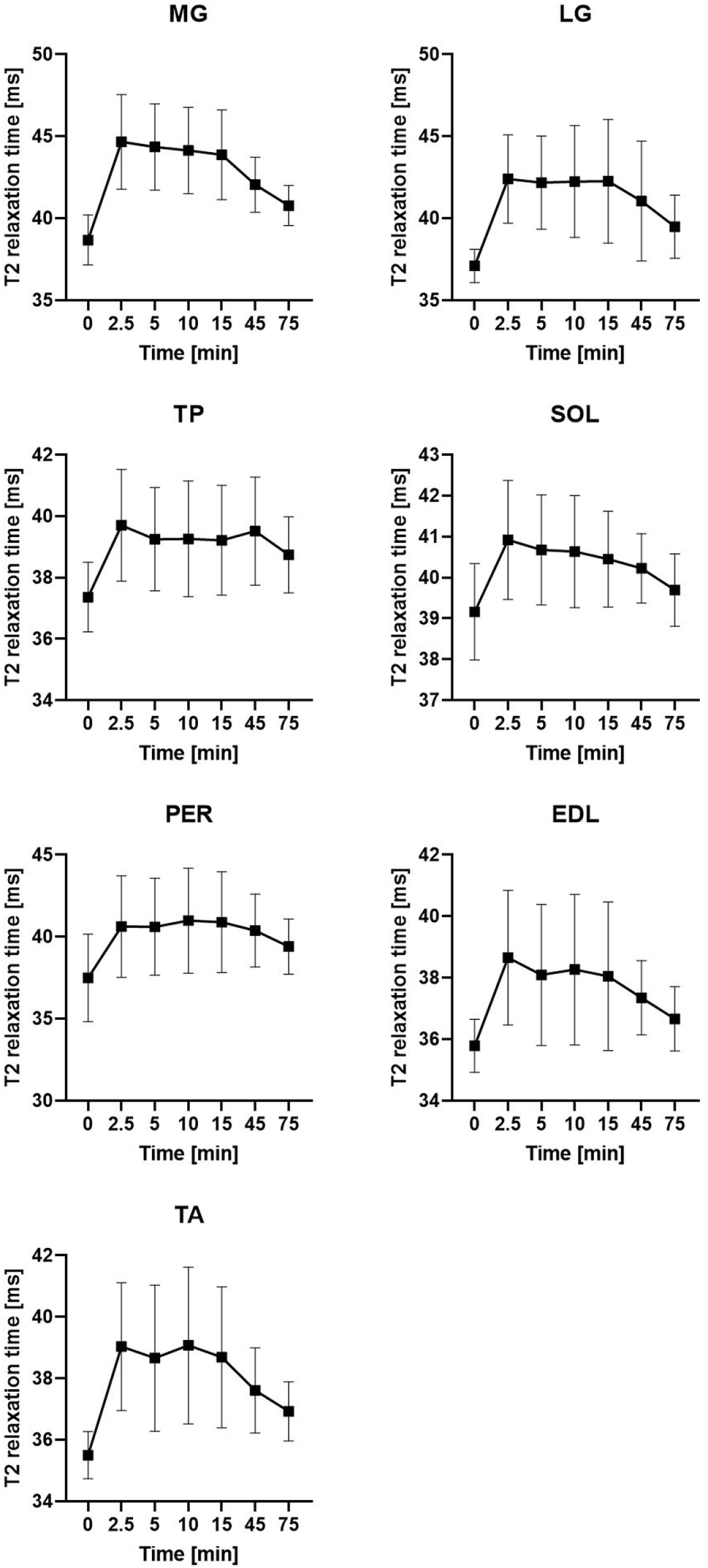
Mean relaxation times with standard deviation for the extrinsic foot muscle.

**Figure 3:**
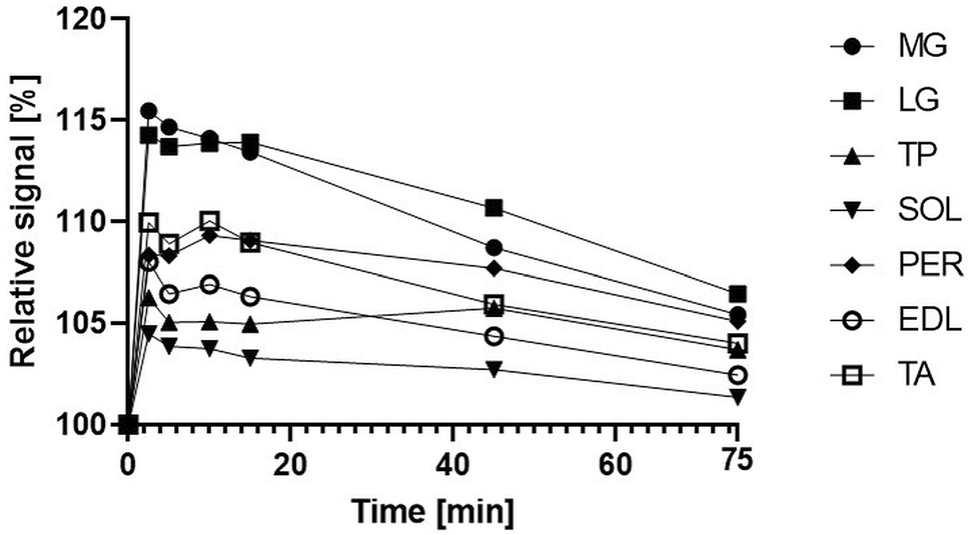
Relative changes of all muscles over time.

**Figure 4:**
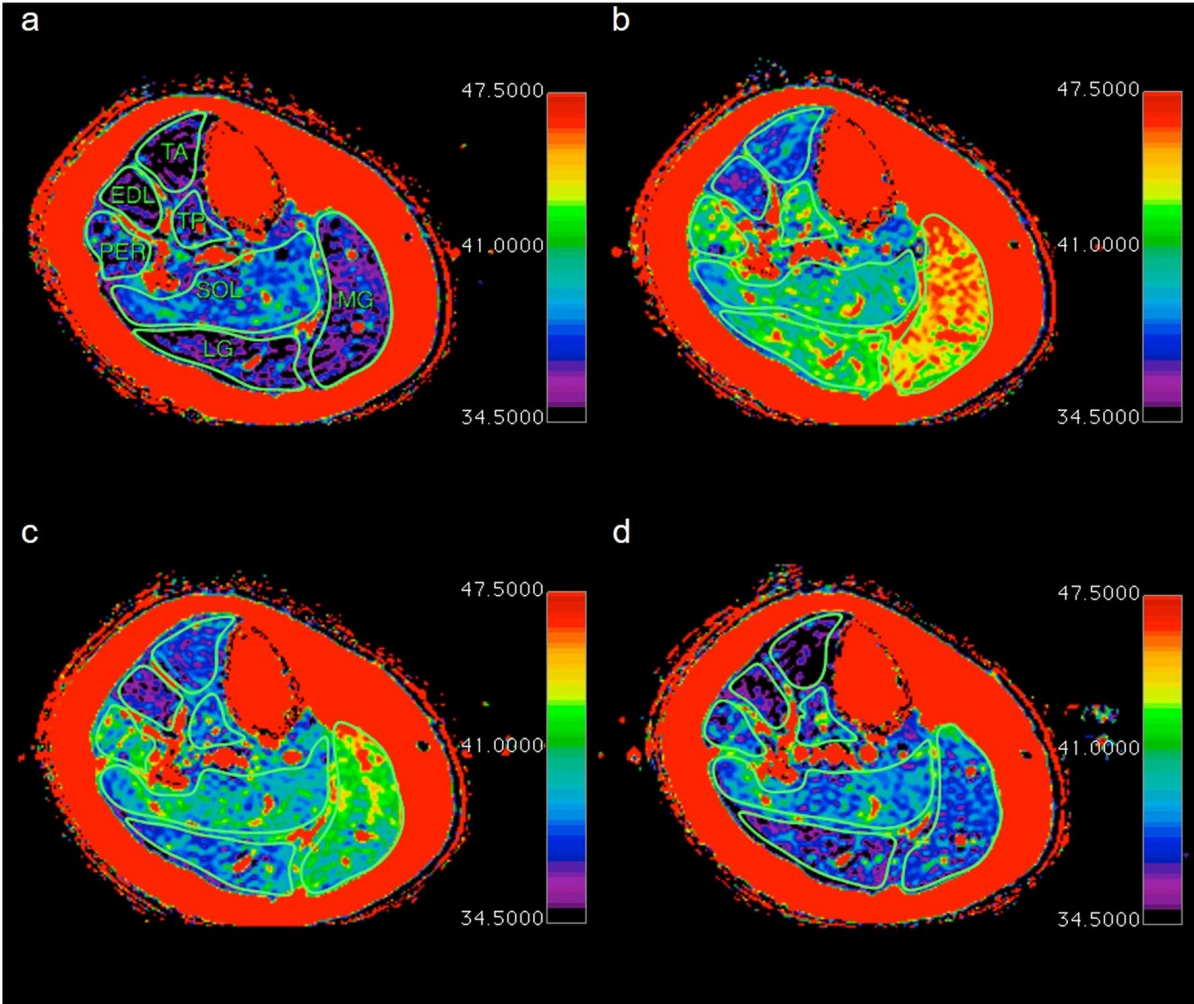
Time course for the relaxation times with the initial increase between baseline (**a**) and 2.5 min (**b**) with the following plateau tille 15 min (**c**) and the later decrease at 75 min (**d**).

The initial ANOVA showed statistical significance for all muscles with R squared ranging from 0.80 to 0.45 (MG: 0.80; LG: 0.78; TP: 0.49; SOL: 0.45; PER: 0.63; EDL: 0.55; TA: 0.58). All segmented muscles showed a statistical significant increase of relaxation times between baseline and 2.5 min (MG: + 15.48% ; LG: + 14.28%; TP: + 6.27%; SOL: + 4.49%; PER: + 8.36%; EDL: + 8.02%; TA: + 9.95% (all *p* < 0.01)) (Table 2) (Fig. 2). The initial steep increase was followed by a plateau phase, including time points 2.5, 5, 10, and 15 min. In this phase, there were no significant differences between any of these time points (all *p* > 0.05) except for the EDL with a decline of T2 relaxation times from 38.66 to 38.10 ms (*p* = 0.03) between 2.5 and 5 min. The initial increase and plateau were followed by reduced relaxation times in the later running phases (Fig. 3). As a result, the relaxation times of all muscles were significantly lower at 75 min than for at least one previous timepoint (all *p* < 0.05) (Fig. 4). MG, LG, SOL, and TA showed significantly higher relaxation times for all time points during the plateau phase (2.5–15 min) compared to the 75 min time point (all *p* < 0.05). The plateau phase was extended for TP and PER without any significant difference between 2.5 and 45 min (all *p* > 0.05), while both muscles showed a significant decrease from 45 to 75 min (TP: *p* = 0.01; PER: *p* = 0.02).

**Table 2:** Absolute and relative changes of the T2 relaxation times during prolonged running. */* marking significant difference (*p* < 0.05) compared to baseline (0 min) (* before diagonal slash) or 75 min timepoint (* after diagonal slash).

	0 min	2.5 min	5 min	10 min	15 min	45 min	75 min
MG [ms]	38.68 ± 1.52 (100%)–/*	44.67 ± 2.89 (115.48%)*/*	44.36 ± 2.63 (114.68%)*/*	44.14 ± 2.63 (114.11%)*/*	43.88 ± 2.74 (113.44%)*/*	42.06 ± 1.68 (108.73%)*/*	40.79 ± 1.22 (105.44%)*/–
LG [ms]	37.10 ± 1.01 (100%)–/*	42.40 ± 2.69 (114.28%)*/*	42.25 ± 2.84 (113.70%)*/*	42.25 ± 3.40 (113.88%)*/*	42.27 ± 3.78 (113.92%)*/*	41.06 ± 3.65 (110.67%)*/–	39.50 ± 1.92 (106.46%)*/–
TP [ms]	37.36 ± 1.13 (100%)–/*	39.70 ± 1.82 (106.27%)*/–	39.25 ± 1.69 (105.06%)*/–	39.26 ± 1.89 (105.09%)*/–	39.22 ± 1.79 (104.97%)*/–	39.51 ± 1.76 (105.76%)*/*	38.74 ± 1.24 (103.70%)*/–
SOL [ms]	39.16 ± 1.18 (100%)–/–	40.92 ± 1.46 (104.49%)*/*	40.68 ± 1.34 (103.87%)*/*	40.64 ± 1.37 (103.76%)*/*	40.45 ± 1.17 (103.29%)*/*	40.23 ± 0.85 (102,72%)*/*	39.70 ± 0.89 (101.36%)–/–
PER [ms]	37.49 ± 2.67 (100%)–/*	40.63 ± 3.10 (108.36%)*/–	40.61 ± 2.96 (108.32%)*/–	40.99 ± 3.21 (109.32%)*/–	40.89 ± 3.08 (109.07%)*/–	40.38 ± 2.22 (107.70%)*/*	39.41 ± 1.69 (105.11%)*/–
EDl [ms]	35.79 ± 0.86 (100%)–/*	38.66 ± 2.19 (108.02%)*/*	38.10 ± 2.29 (106.45%)*/–	38.27 ± 2.45 (106.93%)*/*	38.05 ± 2.42 (106.33%)*/–	37.35 ± 1.20 (104.37%)*/*	36.67 ± 1.04 (102.46%)*/–
TA [ms]	35.50 ± 0.77 (100%)–/*	39.04 ± 2.08 (109.95%)*/*	38.66 ± 2.38 (108.90%)*/*	39.07 ± 2.55 (110.05%)*/*	38.69 ± 2.29 (108.97%)*/*	37.61 ± 1.38 (105.94%)*/–	36.93 ± 0.96 (104.02%)*/–

**Figure 5:**
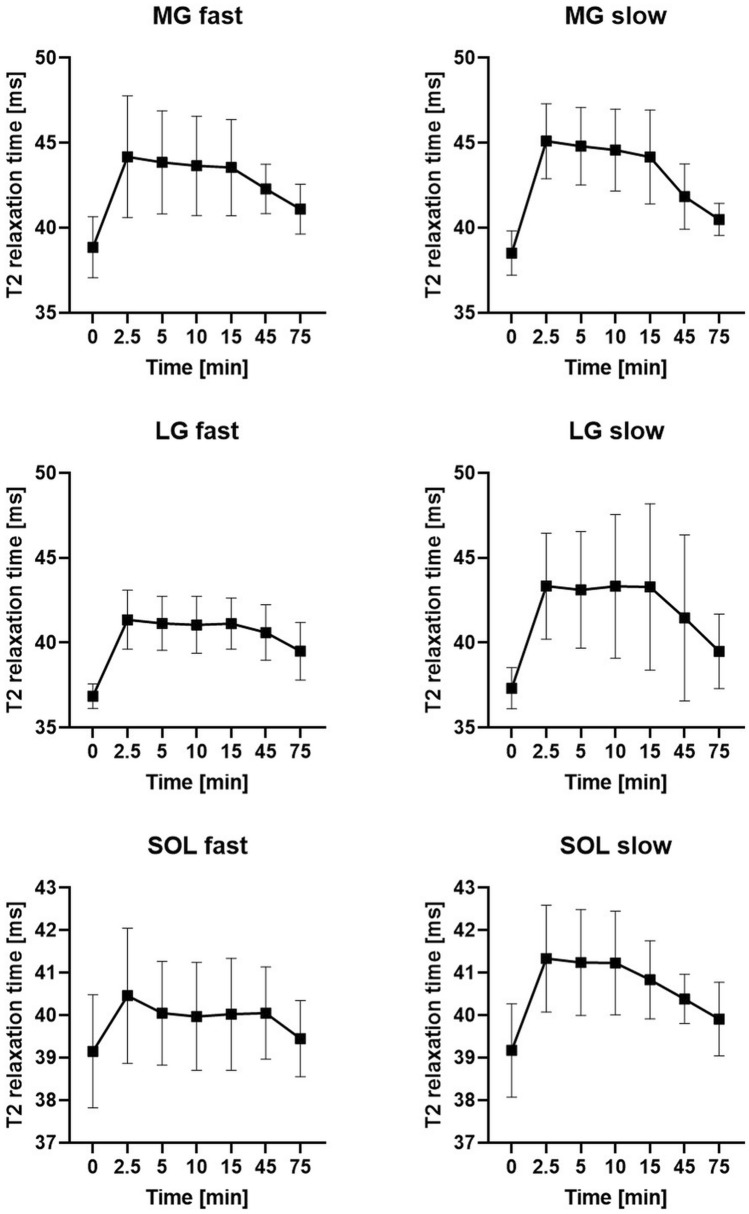
In the fast group, there is initially a smaller increase of the T2 relaxation times and at the same time a less pronounced drop, exemplarily with the plantar flexors MG, LG and SOL.

In the study population, there was a significant negative correlation between running speed as a measure of exercise intensity and the increase of relaxation times for MG (r =  − 0.57, *p* = 0.01), LG (r =  − 0.46, *p* = 0.04) and SOL (r =  − 0.47, *p* = 0.04). For example, the initial increase in MG for the fastest male (13 km/h) and female (11 km/h) were 2.80 ms and 3.33 ms while they were 7.96 ms (8 km/h) and 8.51 ms (7.5 km/h) for the slowest male and female, respectively. The correlation coefficients for all other muscles were negative as well but missed statistical significance (TP: r =  − 0.26, *p* = 0.27; PER: r =  − 0.17, *p* = 0.48; EDL: r =  − 0.28, *p* = 0.24; TA: r =  − 0.38, *p* = 0.11). The whole participant collective could be divided into nine faster and ten slower runners using a running speed threshold of 10 km/h (Table 3). The groups differed significantly regarding speed (*p* = 0.01), height (*p* = 0.03) and sex (*p* = 0.01). Based on this subdivision, there was a smaller increase of relaxation times and a smaller decrease in the later phases for the faster compared to the slower runners. Compared to faster runners, in slower runners a significantly higher relaxation time was measured for all muscles (*p* < 0.05), except the PER, at least once before the end of the run (75 min). In contrast, this was only true for EDL (5 min; *p* = 0.04) and TA (2.5 min, *p* = 0.01) for the faster runners at one time point each. The difference was particularly pronounced for MG, LG and SOL (Fig. 5), where at least three of the first four time points had significantly higher relaxation times compared to 75 min in the slower running group (all *p* < 0.05). This was not the case at any timepoint for the faster runners.

**Table 3:** Relative changes for fast and slow runners. The initial increase is lower for the fast runners. At the same time, however, there is also a smaller drop in the later running phases. * marking significant difference (*p* < 0.05) compared to 75 min timepoint (* after diagonal slash).

		2.5 min (%)	5 min (%)	10 min (%)	15 min (%)	45 min (%)	75 min (%)
MG	Fast	113.71	112.87	112.34	112.08	108.84	105.79
	Slow	117.08*	116.32*	115.72*	114.67*	108.63	105.13
LG	Fast	112.22	111.63	111.38	111.58	110.17	107.17
	Slow	116.12*	115.54*	116.09*	116.00*	112.11	105.83
TP	Fast	105.17	103.98	104.55	104.74	105.97	103.87
	Slow	107.25	106.04	105.57	105.17	105.57*	103.55
SOL	Fast	103.35	102.30	102.10	102.23	102.31	100.77
	Slow	105.51*	105.28*	105.24*	104.24	103.08	101.88
PER	Fast	107.42	108.07	108.60	108.86	108.10	106.46
	Slow	109.17	108.53	109.95	109.25	107.35	103.92
EDl	Fast	105.98	104.68*	105.71	105.39	105.19	102.65
	Slow	109.84*	108.04	108.02	107.17	103.64	102.28
TA	Fast	107.71*	106 .87	108.19	108.27	107.18	103.91
	Slow	111.93*	110.69	111.71	109.59	104.83	104.12

## Discussion

Overuse-related running injuries represent a severe problem for the individual runner, recreational or elite level, and the community with high insurance costs and lost workdays. Despite a clear trend with more people running longer distances (e.g., + 31.61% for marathon finishers in the United States between 2004 and 2016^26^), there is a gap of knowledge regarding physiological changes during prolonged running. This study extends the current knowledge about short-term effects of muscle activation of the extrinsic foot muscles (< 15 min) with changes during a 75 min run using the established T2 mapping. This is the first study to prove that T2 relaxation times do not remain at a plateau but significantly decrease already during exercise for all muscles. Additionally, the increase of relaxation times is negatively correlated with the running speed of the participants, and faster runners experience a lower drop of relaxation times during later running phases.

In line with previous studies^19,27,28^, we recognized significantly increased relaxation times after 2.5 min for all measured muscles ranging from 4.49% for the SOL and 15.48% for the MG or 14.28% for LG. While these three muscles work synergistically, the SOL mainly consists of slow-twitching type I muscle fibers^29^ with lower relaxation times^30^ and is always activated to counteract gravity^31^, which explains the longest relaxation time at baseline and smallest increase during exercise in our study. Absolute T2 relaxation times for baseline as well as for the plateau phase are in the range of pre- and post-treadmill exercise results from Varghese et al.^28^, especially when considering magnet-field strength dependency with a 3 T magnet used in this study^32^. Based on these first insights into muscular processes during prolonged running, this newly discovered signal decrease might be a novel, objective marker for physiological adaptation due to osmolyte changes, especially when considering the evidence for a fast increase of blood lactate followed by a plateau phase and decrease between 15 and 30 min^33^. On the other hand, it might indicate muscular fatigue with reduced activation of the extrinsic foot muscles and changed movement patterns, which are known to lead to increased and altered bone strain^34^ and therefore increasing the risk for running-related injuries such as fatigue fractures or tibia anterior syndrome. In line with this, Kellis et al. found increased quadriceps muscle activity after ankle fatigue^35^, and Sanno et al. described a shift of the joint work from distal to proximal during prolonged running with a continuous decrease in joint work at the ankle through the plantar flexors reaching significance at 5 km^36^, a distance completed by our volunteers between 15 and 45 min. Moreover, assuming all other parameters remain constant, a faster running speed results in higher mechanical work^37^. The negative correlation between running speed and signal increase as well as the reduced signal drop for faster runners in the later phases for MG, LG and SOL, therefore, implies either an altered muscle fiber composition and metabolism due to training effects or a more efficient motion pattern for better trained runners resulting in a reduced muscular strain. This would be in line with Costill et al. who found significantly higher rates of type I fibers in long-distance runners^38^ or with Sanno et al. who described smaller shifts of joint work in trained athletes compared to recreational runners^36^ and Chapman et al. with significant longer activations in EMG for the extrinsic foot muscles for less trained compared to elite level runners^39^, respectively.

This study has several limitations especially lacking physiological markers such as intramuscular/venous lactate concentrations, which might have provided evidence for the underlying physiological processes. Accordingly, additional studies should include blood samples and T2 mapping of other muscle groups for further differentiation of the effect. Furthermore, the number of participants was rather small. However, the initial increase of relaxation times, plateau phase, and final decrease were present in all participants, male and female, and reached statistical significance for all muscles. Faster imaging after every running segment might be realized with a MR-compatible treadmill, as Varghese et al.^28^ used, faster positioning of the volunteers in a standardized position or with better acceleration techniques including compressed sensing^40^. However, since the duration of the pause for running, resulting from scanning and changing times, did not show any statistical differences between the individual run segments, these changes should only alter the absolute values for the specific times and should not have any systematic effect. Another potential confounding factor is the sex as it is well established that muscle metabolism differs between male and female runners^41^. We tried to represent both groups in this study but distribution is significantly unequally for the running speed subgroups. Additionally, the movement pattern might be differet between male and female runners which might influence activation of individual muscles. All participants were recreational athletes in various disciplines (soccer, basketball, triathlon, track and field). Accordingly, the results may differ for non-active participants or for specific subgroups with specific loads^42^.

In conclusion, significantly reduced T2 relaxation times during later running phases (≥ 45 min) in all calf muscles provide evidence for either metabolic adoption or muscular fatigue during prolonged exercise after an initial increase and plateau phase. Additionally, faster runners show a lower initial increase and later decrease in relaxation times, which strengthens the hypothesis that relaxation times are not only based on exercise intensity but highly depend on training level and muscle fiber composition as a possible candidate for performance testing.

## Data Availability

The datasets generated during and/or analysed during the current study are available from the corresponding author on reasonable request. Competing intersts. The author Kilian Weiss currently works for Philips Healthcare and Gert-Peter Brüggemann for the running shoe company True Motion Running GmbH. The remaining authors have no potential conflict of interest to declare.
